# The Novel Osteopore® Wedge in Medial Opening Wedge High Tibial Osteotomy: A Technical Note

**DOI:** 10.7759/cureus.86272

**Published:** 2025-06-18

**Authors:** Walter-Soon-Yaw Wong, Hun Yi Koh, Kong Hwee Lee, Hamid Rahmatullah Bin Abd Razak

**Affiliations:** 1 Orthopaedic Surgery, Sengkang General Hospital, Singapore, SGP; 2 Orthopaedic Surgery, Singapore General Hospital, Singapore, SGP; 3 Musculoskeletal Sciences, Duke-National University of Singapore (NUS) Medical School, Singapore, SGP

**Keywords:** 3d-printing, bone wedge, fused-deposition modelling, high tibial osteotomy, honeycomb microstructuring, osteopore®, polycaprolactone, synthetic, β-tricalcium phosphate wedge

## Abstract

Medial opening wedge high tibial osteotomy (MOWHTO) is a highly common procedure for correcting varus malalignment of the knee. The current gold standard for filling this osteotomy gap is autologous bone graft, which is associated with donor site morbidity. Allograft bone graft is also an alternative; however, it is associated with certain disadvantages, such as storage cost, limited supply, and the risk of infection transmission. Consequently, there has been a growing interest in and further developments of synthetic options for filling the osteotomy gap.

We would like to introduce and describe the process of manufacturing and utilizing the novel Osteopore® 3D-printed, honeycomb-structured, polycaprolactone and β-tricalcium phosphate (PCL-TCP) synthetic bone wedge for filling the gap in MOWHTO. This study will also include illustrations of postoperative radiographs and CT scans to showcase bony remodeling over a period of up to 12 months.

In the era of additive manufacturing and the pursuit of more biocompatible materials, the use of a patient-specific 3D-printed, honeycomb-structured, PCL-TCP bone spacer wedge in MOWHTO is promising and may serve as a viable alternative to autograft and allograft bone grafts. It offers the potential to maintain biomechanical stability and facilitate the healing of the osteotomy gap without the associated drawbacks of bone graft.

## Introduction

High tibial osteotomy (HTO) is a joint preservation procedure used to correct the mechanical axis in patients with unicompartmental knee osteoarthritis, with some studies showing reconstitution of the degenerated joint following HTOs [1]. Filling the osteotomy wedge gap with autograft, allograft, or synthetic materials has been shown to improve the mechanical strength of HTO, and this has led to earlier weight bearing and faster rehabilitation [2,3].

Synthetic options for bone wedge fillers include β-tricalcium phosphate (TCP) and hydroxyapatite (HA) or both. More recently, polycaprolactone (PCL) has been explored as an alternative. In isolation, PCL is a good synthetic candidate for a bone wedge in view of its good biocompatibility, slow degradation rate, and lesser acidic breakdown products in comparison to the more conventionally used polyesters [4]. It shows good flexibility and elongation, which has been increasingly used in craniofacial bone repair [5]. However, the drawback of PCL is that it does not possess osteogenic potential to induce bone regeneration and has a slow degradation time of up to three years before complete removal from the body; hence, the reason for the integration of TCP to create PCL-TCP [6]. Early research into PCL-TCP has shown a lower compressive strength compared to cortical bone allografts but quicker bone formation with better time-varying mechanical properties compared with conventional biomaterials [7-13].

The Osteopore wedge exhibits a modulus of elasticity closer to that of cancellous bone, reducing stress shielding and promoting physiological load transmission to surrounding tissue [14]. This contrasts with cortical autografts or allografts, which can be overly stiff, potentially leading to stress concentration and graft-related complications. The honeycomb architecture of the Osteopore wedge also enhances mechanical interlocking with host bone while allowing vascular and cellular infiltration [15]. Its geometry closely mimics cancellous bone, promoting more effective osteointegration. Clinically, these biomechanical properties may contribute to reduced correction loss, particularly in osteotomies, by maintaining alignment during the critical early phases of healing and gradually transferring load as the scaffold resorbs and is replaced by new bone [16]. While long-term comparative trials are ongoing, early clinical reports support its ability to provide sufficient mechanical support without compromising biological integration.

The microstructure of synthetic materials has been further explored, with honeycomb scaffolding being one of the forerunners in providing higher compressive strength relative to its weight and porosity compared to the grid structure. 3D printing of a honeycomb structure of bone scaffolds has the intention of mimicking the natural bone structure with equidistant equilateral triangles and porosity. It has shown promising early results of favorable cell proliferation and quickened bone tissue regeneration [9,17-22].

Lastly, HTOs require high precision, and the use of bespoke 3D-printed wedges with accurate dimensions to fill the osteotomy gap can reduce human error. This method has proven advantages in biomechanical accuracy, which can result in a more structurally sound and accurate HTO construct [23].

Combining these three attributes of 3D printing, honeycomb structuring, and a PCL-TCP composition, the novel Osteopore® wedge has shown promising results of higher bone mineralization density versus no-augmentation at six months at an early-stage micro-pig model trial [9]. This technical note describes the surgical technique and early imaging outcomes of the novel Osteopore® wedge in medial opening wedge high tibial osteotomy (MOWHTO).

## Technical report

The general surgical technique with regard to patient selection, indications, and the operative procedure of the MOWHTO has been adapted from Khakha et al. (2021) [24].

Wedge composition

**Figure 1:**
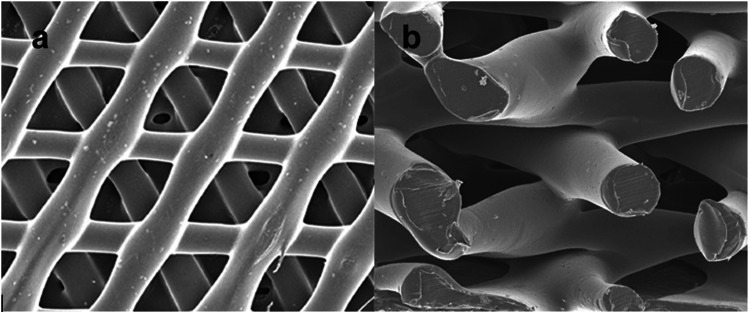
Osteopore® microstructure. a: top view; b: side view.

The synthetic bone wedges were constructed using a blend of PCL-TCP in an 80:20 weight ratio, respectively, and microfilaments ranging in size from 260 μm to 370 μm. This construction process utilized fused deposition modeling (FDM) for 3D printing. FDM was chosen as the additive manufacturing method to create a honeycomb equilateral triangular microstructure with fully interconnected channels, featuring a laydown pattern of 0°/60°/120°. The resulting structure had a porosity of 70% and pore sizing of 0.515 mm (Figure 1). Each layer of the wedge undergoes hydrostatic pressure treatment within a chamber, ranging from 0.01 to 10 atmospheres, while maintaining a temperature just below the melting point of the polymer (between 55°C and 60°C). This process lasts for 30 to 45 minutes to ensure adequate adhesion. The wedges were manufactured in an ISO13485-certified facility and subjected to gamma sterilization. The compressive modulus of the scaffolding is approximately 23.1 MPa, with a compressive strength of 6.38 MPa [9,12,22,25,26]. In comparison, the compressive strength of cancellous bone grafts ranges from 2 to 12 MPa, while that of cortical bone grafts ranges from 130 to 200 MPa. The macro wedge size was customized according to each patient’s needs, as determined by the operating surgeon and as described below.

Preoperative planning

**Figure 2:**
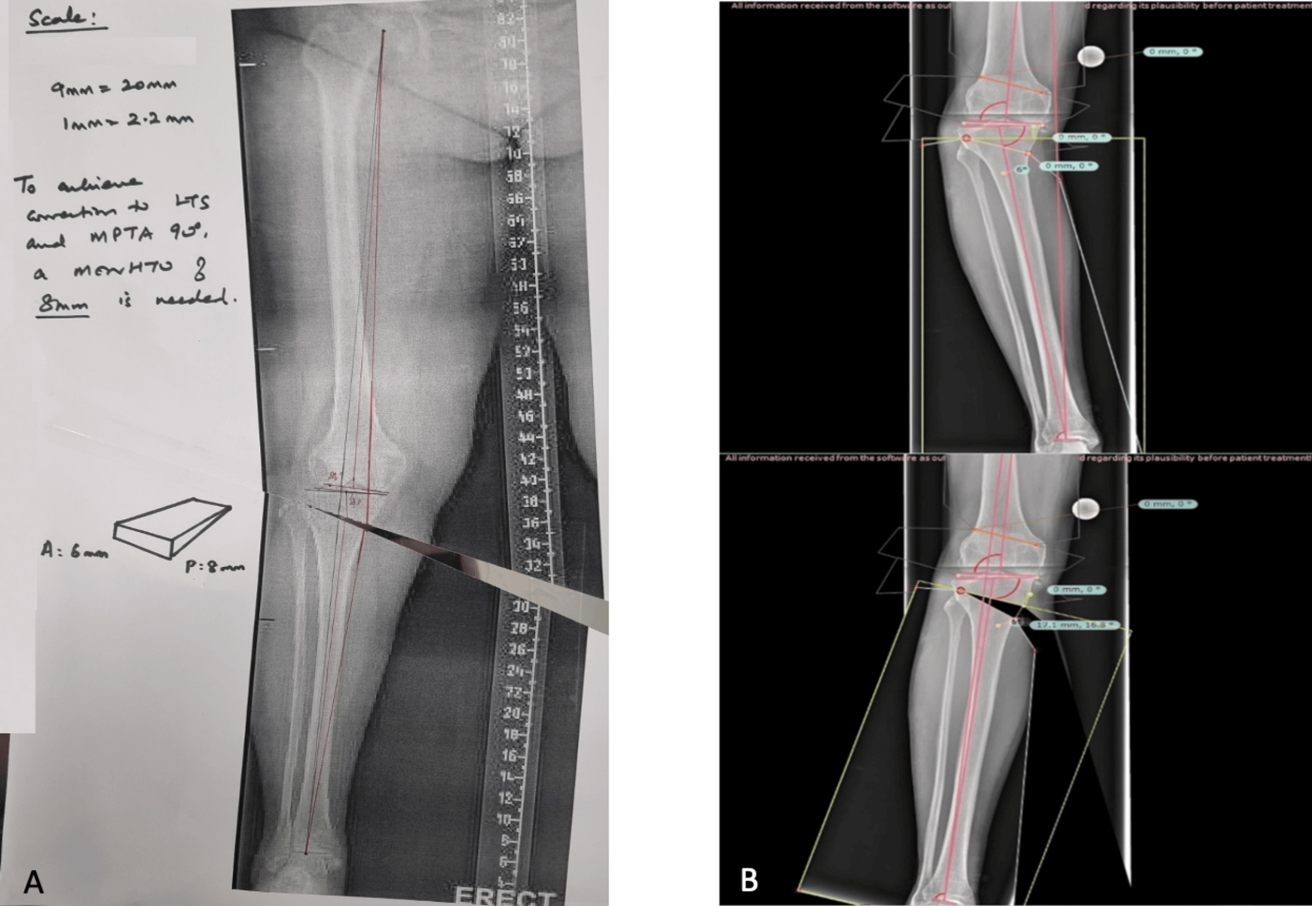
Preoperative planning. A: manually templated high tibial osteotomy; B: digitally templated high tibial osteotomy

**Figure 3:**
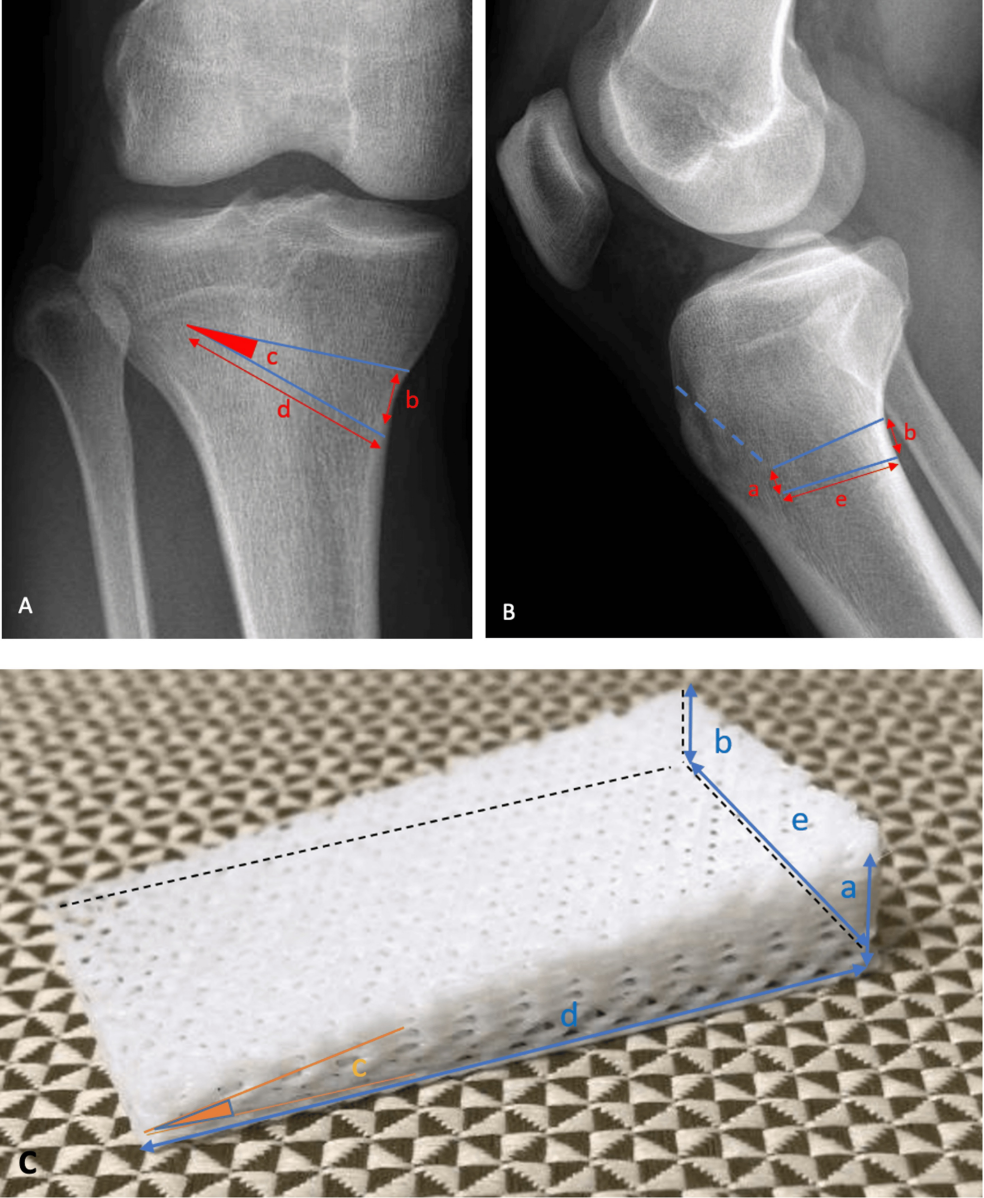
Required dimensions for the bone spacer wedge. A: anteroposterior knee radiograph with the bone wedge dimensions overlayed; B: lateral knee radiograph with the bone wedge dimensions overlayed; C: image of the Osteopore bone wedge used in high tibial osteotomies with labelled dimensions; a: anterior wedge height (conventionally measured as “b-2 mm”; however in anterior cruciate ligament-deficient patients, increased correction may be beneficial), b: posterior wedge height (measured from the medial cortex as the gap required to create the desired mechanical axis correction. We would recommend that 1.2 mm cut be factored in for the thickness of the precision saw blade); c: wedge angle (measured as the lateral most angle of the tibia seen on the anteroposterior radiograph); d: wedge length (measured as the medial to lateral length of the inferior most edge on the anteroposterior radiograph); e: wedge width (measured as 33% to 100% from the anterior cortex (0%) and the posterior cortex (100%) at the pre-designated site of the inferior most edge of the osteotomy on the anteroposterior radiograph).

Preoperative planning was performed either utilizing the Minniaci method using pen and paper or digital planning on TraumaCad® (Figure 2) [27]. When performing MOWHTO for patients with medial compartment knee osteoarthritis and intact lateral compartment, we aim for correction of the mechanical axis to 55% when measured from the medial tibial plateau border (0%) and lateral tibial plateau border (100%). However, in patients with lateral compartment wear (maximum Grade 2), we would recommend for lesser correction of the mechanical axis to reduce the risk of overcorrection of the mechanical axis into valgus, which may lead to poorer patient satisfaction [28]. Following preoperative planning, the following values are required to create the 3D-printed, patient-specific Osteopore® wedge: (a) anterior wedge height, (b) posterior wedge height, (c) wedge angle, (d) wedge length, and (e) wedge width (Figure 3).

Operative procedure

**Figure 4:**
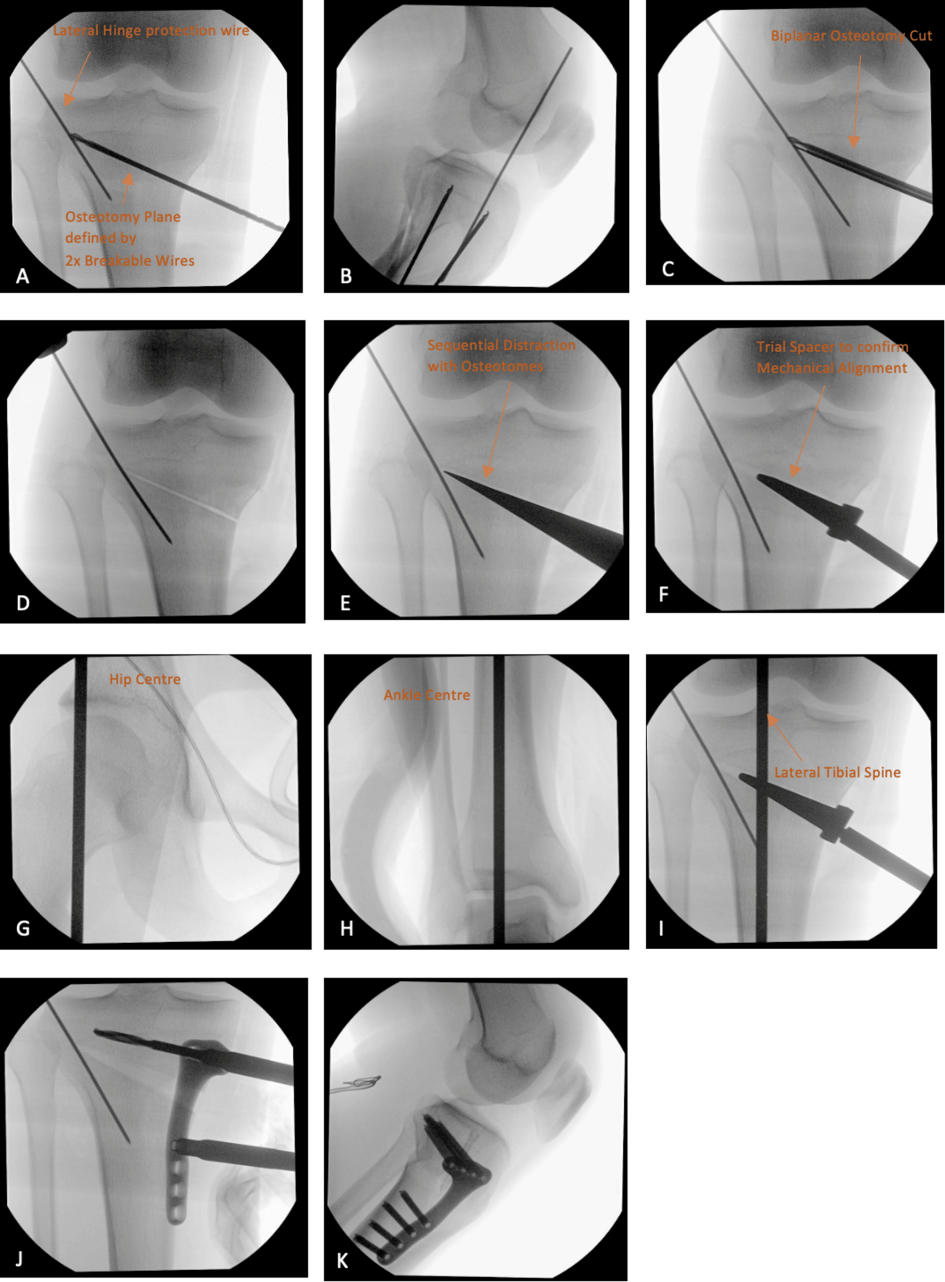
Intraoperative fluoroscopic images of the high tibial osteotomy using the Osteopore® bone wedge. A: anteroposterior view showing the lateral hinge protection wire and osteotomy plane defined by two breakable wires; B: lateral view showing the lateral hinge protection wire and osteotomy plane defined by two breakable wires; C: anteroposterior view showcasing the osteotomy cut being made; D: anteroposterior view showing the intact hinge after the osteotomy cut has been made; E: anteroposterior view sequential distraction with osteotomes; F: anteroposterior view showing a spacer trial being utilized; G-I: mechanical alignment being assessed from the hip center and ankle center, ensuring that it passes through the lateral tibial spine; J: anteroposterior view showing the fixation of the psteotomy plate with the Osteopore bone graft in the osteotomy site; K: lateral view showing the post-fixation of the psteotomy plate with the Osteopore bone graft at the osteotomy site.

The MOWHTO steps are summarized below with the intraoperative fluoroscopic images shown in Figure 4. A longitudinal incision over the medial proximal tibia is performed centered over the pes anserinus. The pes anserinus and the anterior superficial medial collateral ligament are released off their distal attachment and tagged. Two Arthrex® breakable wires are inserted to define the osteotomy plane under fluoroscopic guidance. A temporary protection 1.7 mm K-wire is then drilled from the edge of the lateral tibial plateau into the intramedullary canal to reinforce the lateral hinge [29]. The biplane osteotomy cut is performed using a Stryker® precision saw under fluoroscopic guidance, and breakable wires are subsequently removed. Sequential distraction of the osteotomy gap with osteotomes is then performed. The pre-planned correction is achieved with a metal wedge spacer, and fluoroscopic checks are performed to ensure that the mechanical axis has been restored. The pre-constructed Osteopore® wedge is then press-fit into the osteotomy gap. A good option for reshaping the wedge, if required, is to use a sharp scalpel to make minor adjustments to the shape. A bioabsorbable magnesium screw is inserted into the lateral cortex to further improve the biomechanical strength of the lateral hinge. A final fluoroscopic check is performed to ensure satisfactory alignment. The HTO locking plate and screws are then finally secured followed by the repair of soft tissues before closure.

Postoperative regimen

Following the procedure, patients with no concomitant soft tissue repair and with satisfactory alignment will be put on partial weight bearing as tolerated for four weeks with no restriction on range of motion. Weight bearing is progressed to full at four weeks postoperatively.

Postoperative Radiographs

**Figure 5:**
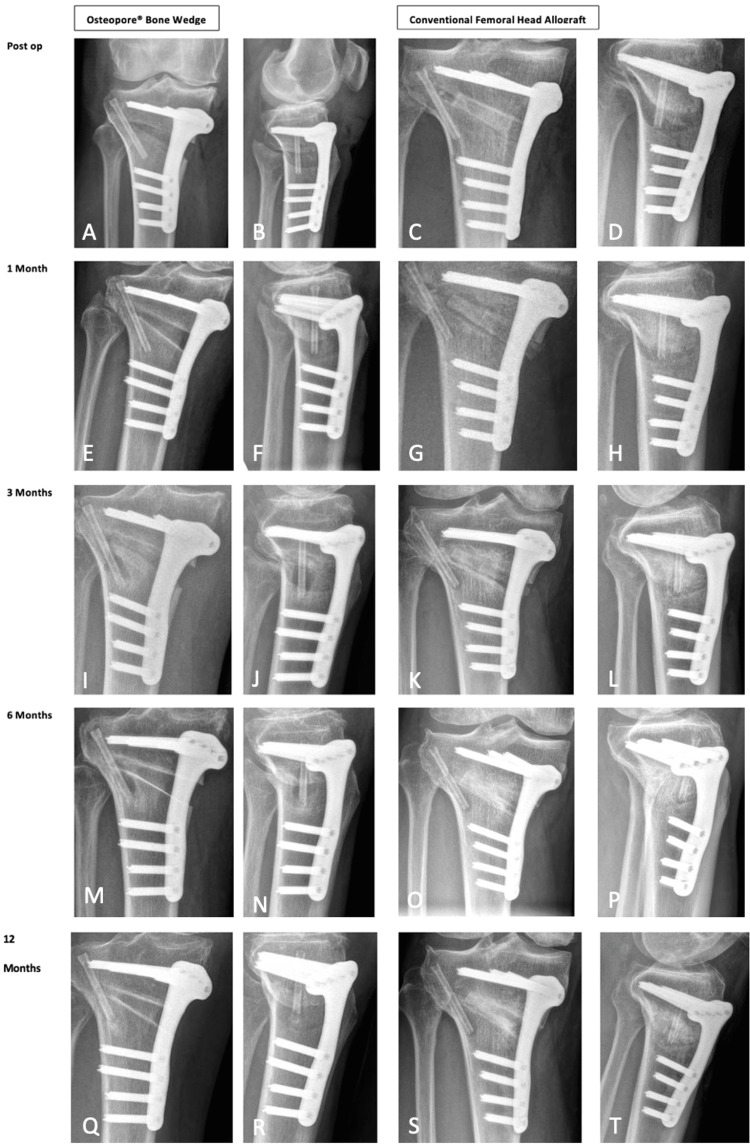
Postoperative radiographs comparing Osteopore® bone wedge and conventional femoral head allograft for up to 12 months. A: anteroposterior view of the Osteopore® bone wedge immediately postoperatively; B: lateral view of the Osteopore® bone wedge immediately postoperatively; C: anteroposterior view of the conventional femoral head allograft immediately postoperatively; D: lateral view of the conventional femoral head allograft immediately postoperatively; E: anteroposterior view of the Osteopore® bone wedge one month postoperatively; F: lateral view of the Osteopore® bone wedge one month postoperatively; G: anteroposterior view of the conventional femoral head allograft one month postoperatively; H: lateral view of the conventional femoral head allograft one month postoperatively; I: anteroposterior view of the Osteopore® bone wedge three months postoperatively; J: lateral view of the Osteopore® bone wedge three months postoperatively; K: anteroposterior view of the conventional femoral head allograft three months postoperatively; L: lateral view of the conventional femoral head allograft three months postoperatively; M: anteroposterior view of the Osteopore® bone wedge six months postoperatively; N: lateral view of the Osteopore® bone wedge six months postoperatively; O: anteroposterior view of the conventional femoral head allograft six months postoperatively; P: lateral view of the conventional femoral head allograft six months postoperatively; Q: anteroposterior view of the Osteopore® bone wedge 12 months postoperatively; R: lateral view of the Osteopore® bone wedge 12 months postoperatively; S: AP view of the conventional femoral head allograft 12 months postoperatively; T: lateral view of the conventional femoral head allograft 12 months postoperatively.

Figure 5 displays postoperative radiographs comparing the use of the Osteopore® wedge and femoral head allograft over 12 months. A key distinction is the radiodensity between Osteopore® and femoral head allograft bone wedges. Initially, Osteopore® exhibits greater radiolucency than the surrounding tibial bone in immediate postoperative radiographs. Over time, a distinct sclerotic line forms along the superior and medial endplates of the wedge, as observed in the anteroposterior radiographs. The sclerotic line likely represents bone remodeling at the wedge-bone interface, reflecting stress adaptation or osteointegration. We would suggest that future studies evaluate this and correlate it clinically. Notably, the development, continuity, and alignment of bony struts between the Osteopore® wedge and surrounding tibial bone become more pronounced, particularly from month six onwards. This alignment potentially indicates remodelling and osteointegration, mimicking the biomechanical forces experienced by the native tibial cancellous bone. The radiolucency of the Osteopore® wedge gradually diminishes over time, suggesting a form of creep substitution bone healing, contrasting with the callus formation commonly seen in conventional femoral head allograft bone wedges.

Postoperative Computed Tomography

**Figure 6:**
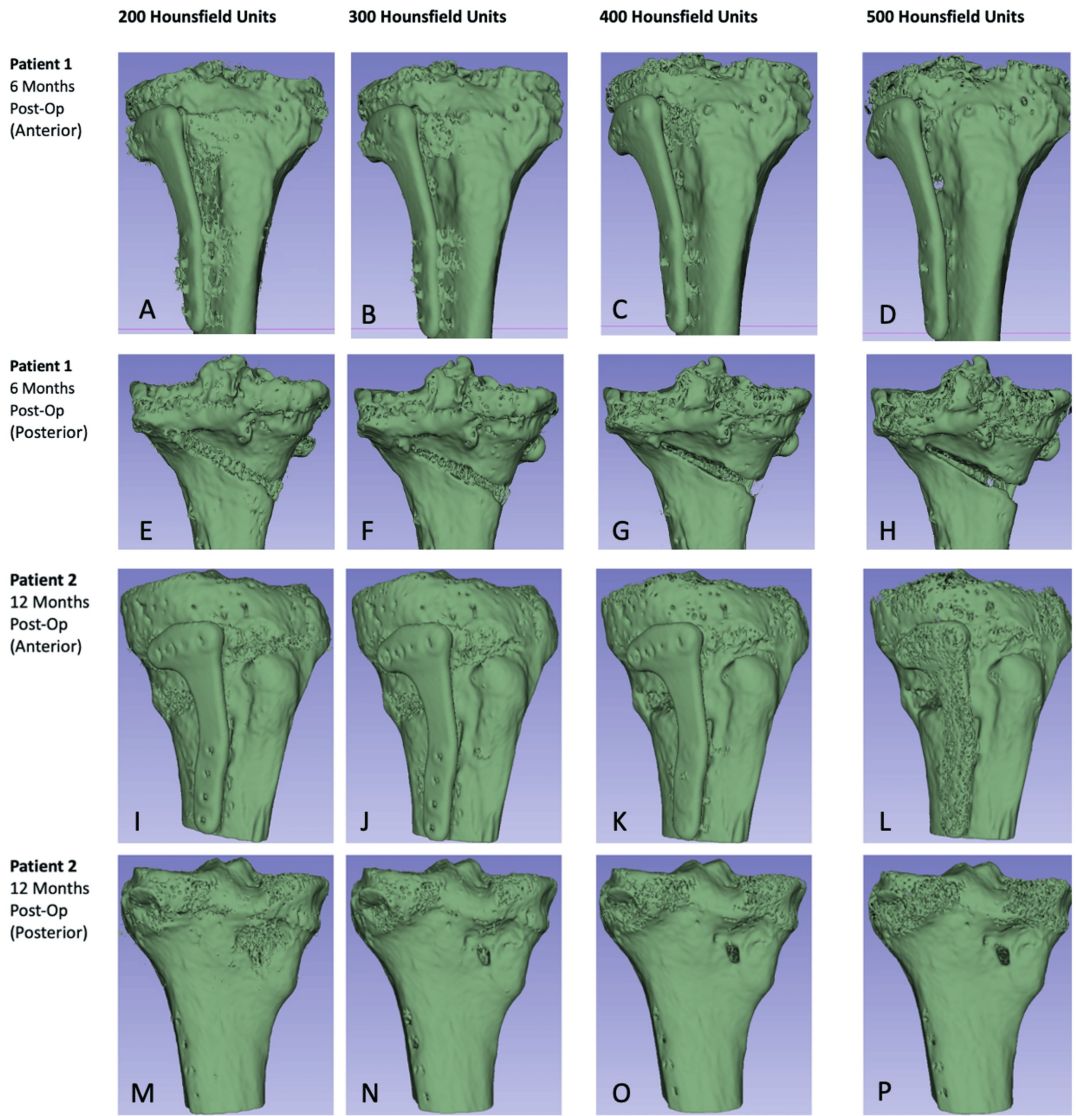
Postoperative CT 3D reconstruction scans at 6 and 12 months. A: six months postoperative anterior view of CT 3D reconstruction at 200 HU; B: six months postoperative anterior view of CT 3D reconstruction at 300 HU; C: six months postoperative anterior view of CT 3D reconstruction at 400 HU; D: six months postoperative anterior view of CT 3D reconstruction at 500 HU; E: six months postoperative posterior view of CT 3D reconstruction at 200 HU; F: six months postoperative posterior view of CT 3D reconstruction at 300 HU; G: six months postoperative posterior view of CT 3D reconstruction at 400 HU; H: six months postoperative posterior view of CT 3D reconstruction at 500 HU; I: 12 months postoperative anterior view of CT 3D reconstruction at 200 HU; J: 12 months postoperative anterior view of CT 3D reconstruction at 300 HU; K: 12 months postoperative anterior view of CT 3D reconstruction at 400 HU; L: 12 months postoperative anterior view of CT 3D reconstruction at 500 HU; M: 12 months postoperative posterior view of CT 3D reconstruction at 200 HU; N: 12 months postoperative posterior view of CT 3D reconstruction at 300 HU; O: 12 months postoperative posterior view of CT 3D reconstruction at 400 HU; P: 12 months postoperative posterior view of CT 3D reconstruction at 500 HU.

Routine computed tomography (CT) is not typically necessary to assess osteotomy union. However, two separate patients provided informed consent for a more thorough evaluation of Osteopore® wedge union at 6 months and 12 months. Figure 6 illustrates a computer-generated 3D reconstruction of the proximal tibia, ranging from 200 HU to 500 HU, at 6 and 12 months post-operation. The reported range for cancellous/trabecular bone is 100-200 HU, while cortical bone starts from 300 HU [30,31]. The CT scans at 6 and 12 months exhibit a noticeable difference. At six months, primarily at 200 HU, the tissue density in the osteotomy gap resembles that of cancellous bone and replicates its natural trabecular microstructure. However, higher HU levels indicate an apparent absence of cortical bone formation compared to the surrounding tibial cortex, seen most clearly posteriorly. In contrast, the CT at 12 months demonstrates bone formation across the previous osteotomy gap both anteriorly and posteriorly, which seems representative of the development of a neo-cortex with HU values like the surrounding tibial cortex, even at 500 HU.

## Discussion

This technical note is written about the novel Osteopore® 3D-printed, honeycomb-structured, PCL-TCP wedges in MOWHTO. This new technique encompasses three key advancements not conventionally used in HTOs: 3D printing, a combination of PCL-TCP, and honeycomb microstructuring. We have summarized our pearls and tips in Table 1.

**Table 1:** Pearls and tips for the usage of the Osteopore® bone wedge. PCL-TCP: polycaprolactone and β-tricalcium phosphate

Pearls and tips for the usage of the Osteopore® bone wedge
Accurate preoperative planning, either on pen and paper or digital planning software, is paramount to printing a patient-specific Osteopore® 3D-printed PCL-TCP wedge
In patients where the lateral compartment cartilage status and the target of correction are not known preoperatively, it is advisable to print wedges of varying sizes preoperatively
In cases where the wedge needs to be altered to fit the osteotomy, a sharp scalpel can be used to cut the wedge
The authors routinely pre-soak the wedge in vancomycin to reduce the risk of surgical site infection, similar to when they use femoral head allografts

Our experience of using these novel bone wedges, particularly from a 3D printing perspective, comes with certain benefits. First, by using these “pre-fitted” wedges, there was an anecdotal reduction of at least 10 minutes of intraoperative time, as these did not have to be fashioned intraoperatively. Second, we did find that the fit of these 3D-printed wedges tended to be more precise, especially when reviewed in postoperative radiographs compared to the conventional manual fashioning of the wedges (Figure 5). However, further studies, particularly in MOWHTOs, are needed to conclude if 3D-printed or bespoke bone wedges have any clinical superiority over conventional hand-fashioning approaches.

Filling of the osteotomy gap in MOWHTO is controversial, with a large meta-analysis by Han et al. (2015) reporting no significant differences in outcomes for the use of any graft type against no graft [32]. However, many surgeons have still opted to utilize grafts of various material types in view of improving the biomechanical strength of the construct [2,33]. Autograft bone has been the gold standard; however, in view of donor site morbidity, allograft and synthetic options have also been explored [34]. Allograft bone does come with certain disadvantages, such as storage cost, limited supply, and risk of transmission of infection [35]. A novel composite of PCL-TCP alongside honeycombed microstructuring has shown superior compressive strength and biocompatibility compared to conventional synthetic bone scaffolds [8-13]. This could potentially bridge the gap between synthetic bone wedges and autologous bone grafting without the drawbacks of allograft bone.

In our experience, we have found that in postoperative radiographs, Osteopore® wedges tend to be much more distinct in appearance than femoral head allograft bone wedges. This may prove useful in visualizing the osteotomy gap and ensuring there is no correction loss. In two of our patients, CT done at 6 and 12 months showed restoration of cancellous bone bridging at 6 months and more so at 12 months alongside neo-cortex formation (Figure 6). One might argue that the use of allograft bone shows more radiodensity in the osteotomy gap on radiographs compared to the Osteopore® wedge. This is likely related to the type of healing that occurs at the osteotomy gap. While we are looking to investigate this further, we postulate that there is contact healing without callous formation with the use of the Osteopore® wedge as opposed to creeping substitution seen in allograft incorporation, where there is slow, near-complete resorption of the graft with simultaneous deposition of new, viable bone. This is further proven by the fact that the CT reconstruction of the osteotomy healing seen with the Osteopore® wedge is seen at 200 HU, which is very similar to that of native cancellous bone. This shows that the Osteopore® wedge has a similar Young’s modulus to the native cancellous bone.

Another novel advancement in the field of additive manufacturing worth investigating is the clinical advantages of various coatings on synthetic bone scaffolds to enhance their bio-integration. Mesenchymal stem cells (MSCs), when used with PCL-TCP in an animal model, have shown that PCL-TCP is an appropriate scaffold for MSCs to aid in bony regeneration [36]. Homogenized collagen coating for PCL-TCP scaffolds is also another method that has been trialled in vitro, which showed significantly higher water absorption, protein absorption, and bioactivity compared to non-coated samples [37]. Another coating worth investigating further is bone marrow aspirate concentrate, which has shown early postoperative benefits in patients undergoing HTOs [38].

Despite its innovative design, the Osteopore® wedge has limitations. Its lower mechanical strength compared to cortical grafts may restrict use in high-load applications, and its performance in complex or large defects remains understudied. Additionally, as a synthetic scaffold, it lacks intrinsic osteogenic cells and growth factors, which may affect early healing. As it is relatively new, its cost-effectiveness compared to traditional grafts has yet to be fully established.

Overall, the literature on the topic, particularly regarding HTOs, is highly limited, and further studies will be required to evaluate its effectiveness.

## Conclusions

In the era of additive manufacturing and the pursuit of more biocompatible materials, the use of a patient-specific, 3D-printed, honeycomb-structured PCL-TCP bone spacer wedge in MOWHTO is promising and may serve as a viable alternative to autograft and allograft bone grafts. It offers the potential to maintain biomechanical stability and facilitate the healing of the osteotomy gap without the associated drawbacks of bone graft.
